# Optimization of the Preparation of Magnetic Liposomes for the Combined Use of Magnetic Hyperthermia and Photothermia in Dual Magneto-Photothermal Cancer Therapy

**DOI:** 10.3390/ijms21155187

**Published:** 2020-07-22

**Authors:** Anilkumar T. S., Yu-Jen Lu, Jyh-Ping Chen

**Affiliations:** 1 Department of Chemical and Materials and Materials Engineering, Chang Gung University, Kwei-San, Taoyuan 33302, Taiwan; anil24march@gmail.com; 2 Department of Neurosurgery, Chang Gung Memorial Hospital, Linkou, Kwei-San, Taoyuan 33305, Taiwan; alexlu0416@gmail.com; 3 Department of Plastic and Reconstructive Surgery and Craniofacial Research Center, Chang Gung Memorial Hospital, Linkou, Kwei-San, Taoyuan 33305, Taiwan; 4 Research Center for Food and Cosmetic Safety, Research Center for Chinese Herbal Medicine, College of Human Ecology, Chang Gung University of Science and Technology, Taoyuan 33302, Taiwan; 5 Department of Materials Engineering, Ming Chi University of Technology, Tai-Shan, New Taipei City 24301, Taiwan

**Keywords:** magnetic nanoparticles, liposomes, magnetic hyperthermia, photothermal therapy, nanomedicine

## Abstract

In this work, we aimed to develop liposomal nanocomposites containing citric-acid-coated iron oxide magnetic nanoparticles (CMNPs) for dual magneto-photothermal cancer therapy induced by alternating magnetic field (AMF) and near-infrared (NIR) lasers. Toward this end, CMNPs were encapsulated in cationic liposomes to form nano-sized magnetic liposomes (MLs) for simultaneous magnetic hyperthermia (MH) in the presence of AMF and photothermia (PT) induced by NIR laser exposure, which amplified the heating efficiency for dual-mode cancer cell killing and tumor therapy. Since the heating capability is directly related to the amount of entrapped CMNPs in MLs, while the liposome size is important to allow internalization by cancer cells, response surface methodology was utilized to optimize the preparation of MLs by simultaneously maximizing the encapsulation efficiency (EE) of CMNPs in MLs and minimizing the size of MLs. The experimental design was performed based on the central composite rotatable design. The accuracy of the model was verified from the validation experiments, providing a simple and effective method for fabricating the best MLs, with an EE of 87% and liposome size of 121 nm. The CMNPs and the optimized MLs were fully characterized from chemical and physical perspectives. In the presence of dual AMF and NIR laser treatment, a suspension of MLs demonstrated amplified heat generation from dual hyperthermia (MH)–photothermia (PT) in comparison with single MH or PT. In vitro cell culture experiments confirmed the efficient cellular uptake of the MLs from confocal laser scanning microscopy due to passive accumulation in human glioblastoma U87 cells originated from the cationic nature of MLs. The inducible thermal effects mediated by MLs after endocytosis also led to enhanced cytotoxicity and cumulative cell death of cancer cells in the presence of AMF–NIR lasers. This functional nanocomposite will be a potential candidate for bimodal MH–PT dual magneto-photothermal cancer therapy.

## 1. Introduction

Although various treatment modalities are available for cancers, each method has certain limitations. On the other hand, the unique multifunctional features provided by nanoparticles may overcome the efficacy limitations from current cancer therapies and open up new avenues of treatment, as has been shown recently with the rising field of nanomedicine in cancer therapy [1]. One of the examples of nanomedicines for the therapeutic treatment of cancer is to employ energy-absorbing nanoparticles for hyperthermia- or heat-mediated therapy [2]. By focusing energy from an external source, nanoparticles can act as a heat source intratumorally and lead to the death of cancer cells [3,4]. In hyperthermia therapy, the tumor cell destruction can be achieved through apoptosis or necrosis with temperatures higher than 43 °C [5,6]. By acting as a remotely controllable local heat mediator without profoundly affecting the physiological temperature of surrounding healthy tissues, hyperthermia provided by nanoparticles could lead to tumor ablation by shrinking tumors through destroying tumor cells or damaging proteins and structures within cancer cells [7].

Specifically, hyperthermia can be classified into two categories, depending on the type of stimulus used for triggering temperature rise: magnetic hyperthermia (MH), which exploits magnetic nanoparticles (MNPs) within an alternating magnetic field (AMF) [8]; and photothermia (PT), which uses light-absorbing plasmonic nanoparticles activated by a near-infrared (NIR) laser for photothermal therapy [9]. To explore the potential of merging both modalities in order to reach an optimized therapeutic efficacy, the combination of MH and PT represents a novel approach for producing cumulative heating effect [10]. Another rationale for this combined strategy beyond the cumulative effect is based on the complementarity of MH and PT to achieve a synergic effect. Indeed, although the main advantage of MH is that it can be performed without depth limitation, it provides a heating yield (per nanoparticle) much inferior to that of PT. Nonetheless, while PT is more efficient in this aspect, it suffers from a limitation related to the light penetration depth in the tissue [11]. Therefore, nanocomposites that could combine MH and PT, with responsiveness to both magnetic field (AMF) and light (NIR laser) sources, could be suggested as promising candidates to merge the strengths of both cancer therapy modalities while overcoming their intrinsic limitations.

Endowed with phenomenal properties such as satisfactory tunable size, surface chemistry, and biocompatibility, MNPs have gained importance in cancer therapies [12]. Iron oxide MNPs are trending in clinical use for treatment of cancers by direct infusion of MNPs to solid tumors for MH treatment of glioblastoma and prostate carcinoma [13]. Furthermore, since MNPs can be driven to a precise targeted site with an external magnetic field, MNPs alone [14] or nanocomposites containing MNPs [15] are widely exploited for targeted drug and gene delivery. Endeavors are also being made to upgrade the heating proficiency of iron oxide MNPs for tumor ablation. Very recent works showed evidence that iron oxide MNPs, in addition to commonly used plasmonic gold nanoparticles, could be also used for PT by exciting their lattice photons in the NIR region [16]. While a complete understanding of the associated physical phenomena is still under investigation, the use of iron oxides MNPs for PT is under development, but with relatively less reports in the literatures than for MH. As in combination cancer therapy, supplementation of MNP-based MH with other forms of cancer therapy such as PT is deemed advantageous to enhance the therapeutic outcomes and decrease the dosage of MNPs required for MH. Along this line, a recent study used AMF and an 808 nm NIR laser to amplify the heating efficiency of cube-shaped iron oxide MNPs for bimodal magneto-photothermal cancer therapy [17]. A similar study also reported the combined use of AMF and a 532 nm laser treatment to enhance the MH–PT cancer cell killing effects induced by iron oxide MNPs [18].

On the other hand, amphiphilic lipid molecules could be self-assembled into liposomes as either unilamellar or multilamellar vesicles. With a unique design consisting of an aqueous core and a lipid bilayer membrane, liposomes can carry both hydrophilic and hydrophobic drugs. Through encapsulating MNPs in liposomes, magnetic liposomes (MLs) offer multiple functions for biomedical applications, such as magnetic resonance imaging, photoacoustic tomography, and NIR absorption [19]. Specifically, there is an emerging trend in using MLs for cancer treatment by targeted drug and gene delivery and localized hyperthermia [4,20]. Other than the tunable trigger release offered by MLs, one advantage of MLs over MNPs for cancer therapy stems from the targeting capabilities of MLs, either through magnetic targeting or biological targeting [21]. Considering passive targeting of liposomes [22], several studies have shown that cationic liposomes are more efficient as vehicles for drug delivery than neutral or anionic liposomes, possibly due to the electrostatic interactions between the cationic liposomes and the negatively charged cell membranes, enhancing nanoparticle uptake [23,24]. In addition, cationic liposomes can be easily fused with cell membranes to facilitate intracellular uptake [25]. Recent studies indicate that cationic liposomes interact with the endothelial cells of microvessels and lead to a propensity for localization in newly formed tumor vessels to potentially enhance antitumor effects [26]. Nonetheless, it should be also noted that MLs may result in a shorter circulation time due to rapid clearance from the circulation by the reticuloendothelial system, originating from nonspecific interactions of their positive surface charge with anionic species in the blood, as well as a reduction of the enhanced permeability and retention effects [27,28].

In this study, we postulate that a well-designed magnetic liposomal formulation could provide combinatory stimulation of iron oxide MNPs encapsulated in MLs with both AMF and NIR lasers to enhance the heating efficiency, which could overcomes the deficiencies of using stand-alone ML-mediated MH or PT. Toward this end, we encapsulated citric-acid-coated magnetic nanoparticles (CMNPs) inside the aqueous cores of cationic MLs composed of 1,2-dipalmitoyl-sn-glycero-3-phosphocholine (DPPC), dimethyldioctadecyl ammonium bromide (DDAB), cholesterol (CH), and the cationic lipid dimethyldioctadecyl ammonium bromide (DDAB). The first objective of this study was to optimize the preparation of MLs intended for magneto-photothermal cancer therapy. Using response surface methodology (RSM) with the encapsulation efficiency (EE) of CMNPs in MLs and liposome size as the responses, we intend to maximize the EE of CMNPs to enhance the MH–PT efficiency of MLs, while simultaneously minimizing the liposome size to facilitate intracellular uptake. Using the optimized preparation of MLs, the second objective of this study was to characterize the physico-chemical properties, heating efficiency, and cancer cell killing effects toward U87 glioblastoma cancer cells in vitro under single-mode (AMF for MH or NIR laser for PT) or dual-mode (AMF–NIR laser for MH–PT) operation.

## 2. Results and Discussion

### 2.1. Model Development and Optimization

To optimize the preparation of MLs, we chose the lipid composition and CMNP concentration during liposome preparation as the experimental variables and studied the effects on the encapsulation efficiency (EE) of CMNPs and liposome size. By incorporating the experimental design and non-linear regression, those effects were investigated within the context of response surface methodology (RSM), which enables experimental investigation of the interactions of factors simultaneously as opposed to one factor at a time approach. Models of EE and liposome size were constructed, which allow the evaluation of the significance of the parameters and provide the prediction capability for EE and size. The utilization of RSM is a powerful methodology in the enhancement of the optimization of the liposomal preparation within a designed experiment [29,30,31,32]. Based on the statistical theory with 2^5−1^ fractional factorial design, a central composite rotatable design (CCRD) was adopted to design the experiments using four variables and five levels (Table 1). The effects of four independent variables (mass of DPPC, mass of DDAB, mass of CH, and concentration of CMNP, coded separately as X_1_, X_2_, X_3_, and X_4_, respectively) were studied to assess the encapsulation efficiency (EE) of CMNPs and size of MLs. The structure of CCRD comprises 31 experimental runs with seven replicates at the center to estimate the pure error sum of squares, 16 factorial points (−1 and +1), and 8 axial points (−2 and +2). Each experiment in the design was carried out randomly from statistical points of view. The individual experimental condition and their corresponding observed response values from experiments are tabulated in Table 1.

**Figure 1:**
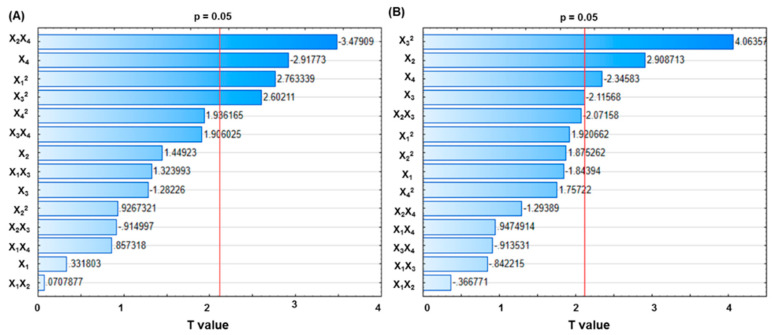
The Pareto charts of standardized effects for the encapsulation efficiency (EE) of CMNPs (**A**) and size of MLs (**B**). The lines in each chart represent the 95% confidence level. Factors with standardized effect values to the right of this line are statistically significant.

The Pareto chart was used to study the effects of variables on EE and size, in which the length of each bar was proportional to the absolute value of the estimated effect (associated regression coefficient) (Figure 1). The vertical line represents the 95% significance limit, which declared statistical significance when the bar associated with a factor crossed this line [32]. From Figure 1A, one cross-product (X_2_X_4_), one linear-coefficient (X_4_), and two quadratic coefficients (X_1_^2^ and X_3_^2^) are found to be significant (*p* < 0.05). Similarly, Figure 1B indicates that one quadratic coefficient (X_3_^2^) and three linear coefficients (X_2_, X_4_ and X_3_) are significant (*p* < 0.05).

For validation of the significance of the RSM model, the trial consequences in Table 1 were applied for the multiple regression analysis and the response surface 3D plots, and predictions over observed values were generated. The fitting of experimental data with a polynomial equation representing the response surface model was used to evaluate the relationship between the factors and the response values statistically. Second-order regression models correlating responses and variables were obtained according to the estimation of data:

Y_1_ = 19.985 + 1.217 X_1_ + 9.855X_1_^2^ + 5.641X_2_ + 3.305X_2_^2^ − 4.991X_3_ + 9.28X_3_^2^ − 11.358X_4_ + 6.905X_4_^2^ + 0.337X_1_X_2_ + 6.312X_1_X_3_ + 4.087X_1_X_4_ − 4.362X_2_X_3_ − 16.55X_2_X_4_ + 9.087X_3_X_4_ (1)

Y_2_ = 127.4 − 11.083X_1_ + 10.576X_1_^2^ + 17.483X_2_ + 10.326X _2_^2^ − 12.716X_3_ + 22.376X_3_^2^ − 14.1X_4_ + 9.676X_4_^2^ − 2.7X_1_X_2_ − 6.2X_1_X_3_ + 6.975X_1_X_4_ + 15.25X_2_X_3_ − 9.5X_2_X_4_ − 6.725 X_3_X_4_ (2)

where X_i_ is the coded value of each variable, Y_1_ is the EE response value, and Y_2_ is the size response value. From the regression results, the R^2^ values are 0.744 for EE and 0.765 for size, which reveal the level of variation of all responses that is predicted by the model. Considering the percentage of variability, the models were used for subsequent prediction of response values during the validation step.

The optimum conditions for preparation of MLs were obtained through regression models in accordance with the limit criterion of the maximum EE response and minimum size response using Statistica software. The optimized conditions estimated by the model equations were DPPC (X_1_) = 2 μmol, DDAB (X_2_) = 0.5 μmol, CH (X_3_) = 1 μmol, and CMNP (X_4_) = 0.25 mg/mL (Table 2). The theoretical predicated values of EE and size under the above conditions were 86.9% and 121.1 nm, respectively. The accuracy of the model was validated with four repeated experiments under aforementioned optimum conditions not employed in the model (Table 2). The experimentally observed values for EE and size were 84.2 ± 4.7% and 124.3 ± 6.5 nm (mean ± SD, *n* = 4), respectively. Therefore, the verification experiments confirmed the validity of the predicted model, with the predicted value being reasonably close to the mean of the experiment value and within its mean ±SD range for both EE and size. Taken together, the results of model validation experiments endorse the model from CCRD as being accurate and reliable for prediction of EE and size.

**Figure 2:**
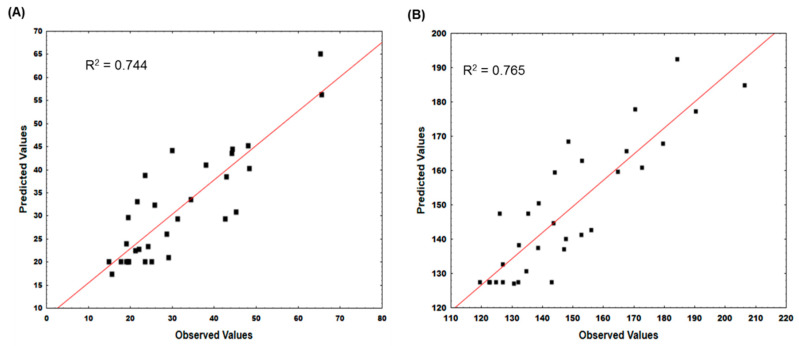
Comparison between the predicted values and the observed values of encapsulation efficiency (EE) of CMNPs (**A**) and size of MLs (**B**).

Further evaluation of the optimization technique was carried out, where the observed value was compared with the observed value for EE (Figure 2A) and size (Figure 2B). The experimental observed values accord well with the predicted values from the model development, where clustering points around the diagonal line confirms a satisfactory correlation between the experimentally observed values and the predicted values. It should be noted that even the model coefficients are empirical, which may not be assigned to any physical or chemical effects. They have been shown to be very useful for predicting outcomes of untested experimental conditions [32].

**Figure 3:**
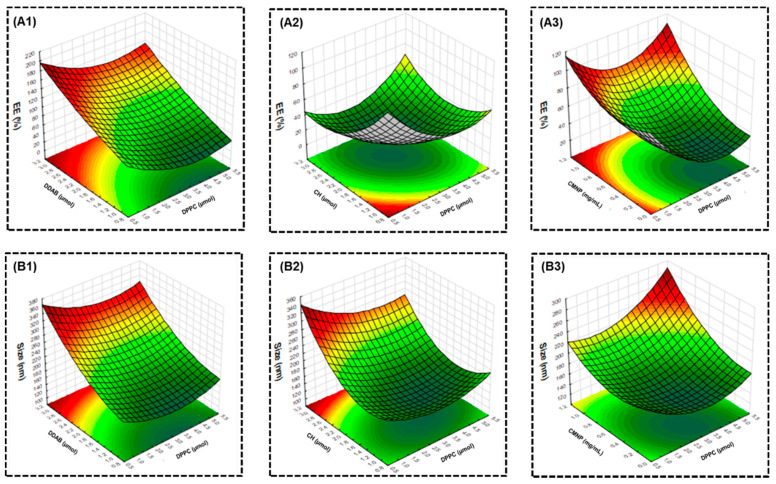
Representative response 3D surface plots showing the effect of factors on the encapsulation efficiency (EE) of CMNPs (**A1**–**A3**) and size of MLs (**B1**–**B3**). The surface 3D contour plots were generated form the response model by varying two of the factors while keeping the other two factors constant, with the variables shown in Table 2.

The relationships between the factors and responses were studied from the surface 3D contour plots by plotting the response model against two of the factors, while keeping the other two factors constant (Figure 3). From Figure 3(A1,B1), the EE and size showed similar dependence, with low DDAB and high DPPC leading to lower response values, but stronger dependence was found for DDAB. From Figure 3(A2,B2), both the low DPPC and CH could maximize EE, while simultaneously minimizing size. For DPPC and CMNPs, low DPPC combined with high CMNPs leads to higher EE. Nonetheless, a small liposome size could only be achieved using low DPPC and low CMNPs (Figure 3(A3,B3)). Overall, the interactions between factors and their influence on the responses support the choice of the optimum formulation from the regression model.

### 2.2. Characterization of Physico-Chemical Properties

**Figure 4:**
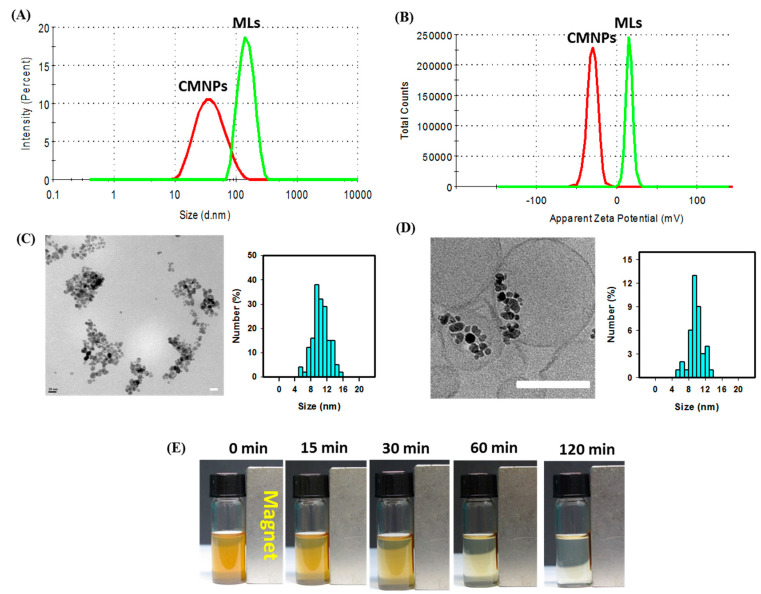
The particle size distribution from the dynamic light scattering (DLS) (**A**) and zeta potential distribution (**B**) of CMNPs and MLs. The TEM image (bar = 20 nm) and histogram of CMNPs (**C**) and the cryo-TEM image of MLs (bar = 100 nm) and histogram of CMNPs in MLs (**D**). (**E**) The magnetic responsiveness of MLs.

The CMNPs were prepared by chemical co-precipitation of Fe^+2^ and Fe^+3^ ions and surface-modified by citrate coating. The MLs were prepared by using the optimum formulation of lipids and CMNPs (Table 2) by the thin film hydration–extrusion method to load CMNPs inside the aqueous cores of the liposomes. The hydrodynamic size distribution was analyzed by dynamic light scattering (DLS) together with the zeta potential values using a Zetasizer (Figure 4A,B). As shown in Table 3, the average particle size (diameter) of CMNPs is 36.5 ± 5.4 nm and for MLs is 124.3 ± 6.5 nm. The polydispersity index (PDI) is below 0.30, providing evidence of uniform particle size distribution in addition to the good suspension stability of MLs (Table 3) [33]. For drug delivery using liposomes, a PDI value of 0.3 or below is considered to be acceptable, which indicates a homogenous distribution of the lipid vesicles [34]. The loading percentage of CMNPs in MLs (weight percentage of CMNPs in MLs) was determined to be 21.8% ± 1.7% (*w/w*). The average zeta potentials are −19.4 ± 4.3 mV and 16.3 ± 3.7 mV from electrophoretic mobility measurements (Table 3). The average zeta potential of CMNPs is negative due to the carboxylate groups of the coated citrate, which changes to a positive value for MLs due to the presence of cationic lipid DDAB in the lipid bilayer. Intracellular uptake will be facilitated by taking advantage of this cationic nature of MLs for targeted magneto-photothermal cancer therapy originated form the MH–PT nature of the CMNPs cargo inside MLs.

The TEM images of CMNPs (Figure 4C) and cryo-TEM imaged of MLs (Figure 4D) show close to spherical morphology, while the particle size is generally consistent with that from DLS measurements. The size of CMNPs was below 20 nm, as assessed by ImageJ analysis of discrete nanoparticles in the agglomerate and shown in the size histogram of CMNPs (Figure 4C). This size is consistent with that observed for the individual CMNPs within the aqueous core enclosed by a lipid bilayer in MLs (Figure 4D). The magnetic responsiveness of MLs was confirmed from the time-lapsed images in Figure 4E, where a 3 mg/mL suspension of MLs in PBS was guided by a magnet to the side of the tube to result in a more transparent solution with time. In general, this behavior will provide additional magnetic targeting ability for MLs through their guidance with an external magnet to the tumor site, followed by MH–PT-induced by AMF–NIR lasers for tumor therapeutic functions [35].

**Figure 5:**
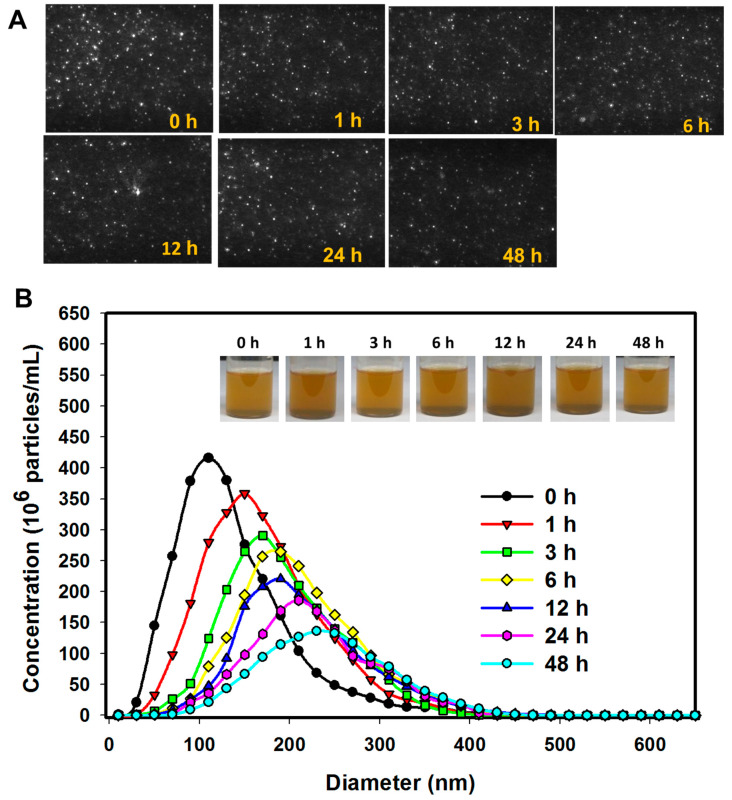
The particle size distribution of MLs in PBS (3 mg/mL) after incubation at 37 °C for different times and subject to nanoparticle tracking analysis (NTA). (**A**) The screenshot images of the original video file of the light scattering particles (MLs) at different times. (**B**) The concentration vs. the diameter of the particles (MLs), with the inserts showing the gross views of the solutions.

The stability of MLs in vitro was determined using nanoparticle tracking analysis (NTA) after incubating MLs in PBS (3 mg/mL) at 37 °C for different durations and tracking the movement of MLs with a 405 nm laser. Using NTA, MLs were observed as scattering points moving under Brownian motion, with larger particles scattering more light and appearing to be bigger [36]. As shown in Figure 5A, no significant change of the size of the scattering light was observed from the screenshot images at any time points. An estimation of the sample polydispersity at any given time due to the high resolution of the captured NTA images also indicated that the sample is fairly monodispersed, which is consistent with the PDI value from the DLS (Table 3). For quantitative comparison, the concentration of particles (MLs) was determined as a function of the particle diameter after different incubation times (Figure 5B). At time 0, the peak particle diameter was close to the value predicted from the RSM model (121 nm) and the experimental value from DLS (124 nm). After incubation for 1 h, the peak particle diameter increased to ~200 nm but the peak particle concentration decreased. Further increase in incubation time did not result in apparent change of peak particle diameter, but the peak particle concentration decreased. No MLs were above 400 nm, even after 48 h incubation. The stability of liposomes depends on many factors, while the shift in particle concentration may be due to destruction of MLs [37]. Particle aggregation may also lead to an increase in size and decrease in counts from NTA analysis [38]. Nonetheless, the remaining MLs of acceptable average size (~200 nm) will not impair the capability for intracellular uptake by cancer cells [39].

**Figure 6:**
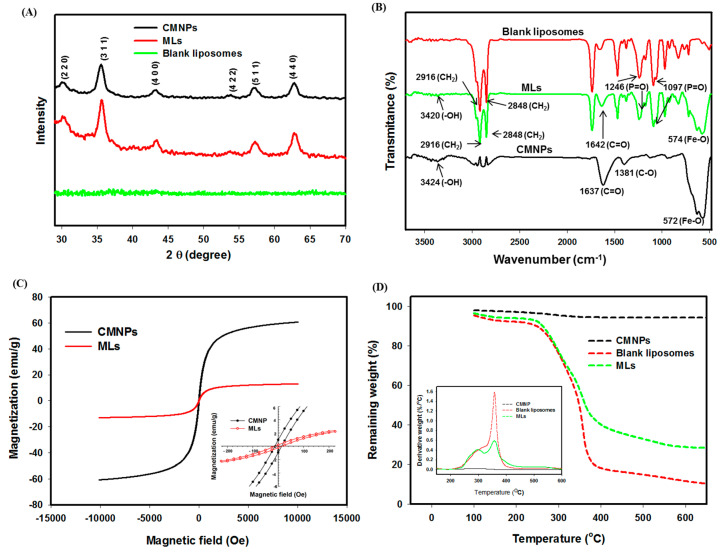
(**A**) Characterization of CMNPs and MLs by X-ray diffraction (XRD) patterns. (**B**) The Fourier transform infrared (FTIR) spectra of CMNPs, MLs, and blank liposomes (MLs without CMNPs). (**C**) The superconducting quantum interference device (SQUID) magnetization curves of CMNPs and MLs, with the insert showing the coercivity and the remnant magnetization. (**D**) Thermogravimetric analysis (TGA) of CMNPs, MLs, and blank liposomes (MLs without CMNPs), with the insert showing the differential thermal analysis (DTA) curves.

We used X-ray diffraction (XRD) to characterize CMNPs, MLs, and blank liposomes (MLs without CMNPs), the diffraction patterns for which are shown in Figure 6A. For CMNPs and MLs, six diffraction peaks were observed at 2*θ* = 30.3°, 35.5°,43.4°, 53.5°, 57.2°, and 62.6°, which could be indexed to the (220), (311), (400), (422), (511), and (440) planes of a cubic cell. The crystalline structure was affirmed to be comparable with that of magnetite (JCPDS card number 19-0629), which indicates pure Fe_3_O_4_ associated with the spinal structure of magnetite in CMNPs [15,32,40]. The absence of the six diffraction peaks in the patterns shown for blank liposomes, which were prepared similarly to MLs but without CMNPs, further supports the encapsulation of CMNPs in MLs. From the strongest diffraction (311) peak, the average crystal grain sizes calculated from the Scherer equation were 14.7 and 17.1 nm for CMNPs and MLs, respectively, by assuming spherical crystals from XRD line broadening, which is consistent with the nanoparticle size of CMNPs observed from cryo-TEM.

Figure 6B shows the Fourier transform infrared (FTIR) spectra of CMNPs, MLs, and blank liposomes (MLs without CMNPs). In CMNPs, the strong absorption peak at 572 cm^−1^ corresponds to the Fe–O bond in the nanoparticles, which also appeared in MLs. The –OH vibrations are confirmed from the peak at 3424 cm^−1^ [41]. The characteristic peaks of citrate at 1394 and 1735 cm^−1^, due to the symmetric vibration and asymmetric stretching of C–O from –COOH group and C=O from –COOH, were shifted to 1381 and 1637 cm^−1^ in CMNPs to successfully support citric acid coating. The encapsulation of CMNPs in MLs could be also confirmed from the absence of the Fe–O stretching bond at 574 cm^−1^ by comparing the spectra of blank liposomes against those of MLs. In both liposomal preparations, characteristic peaks at 1097 cm^−1^ and 1246 cm^−1^ could be assigned to the phosphate group (P=O), while the CH_2_ symmetric and asymmetric stretching provided peaks at 2848 cm^−1^ and 2916 cm^−1^, respectively [42].

Based on superconducting quantum interference device (SQUID) analysis, the magnetization curves determined at room temperature indicate that the saturation magnetization value of CMNPs is 60.5 emu/g (Figure 6C). The saturation magnetization value of MLs is lower than that of CMNPs (13.0 emu/g). The drop in magnetization strength may be associated with encapsulation of CMNPs within the liposomes, as the magnetization sensitivity may decline when the surfaces of CMNPs are occupied by lipids [43,44]. Nonetheless, decreased saturation magnetization may largely stem from the reduced weight percentage of CMNPs in MLs, as the magnetic moment is based on the unit weight of MLs, while all other components in MLs are diamagnetic [45,46]. This could be supported by comparing the expected saturation magnetization value of MLs (13.2 ± 1.0 emu/g), calculated from the loading percentage of CMNPs in MLs (21.8% ± 1.7%), and the value measured using SQUID analysis (13.0 emu/g). The remnant magnetization and coercivity could be determined from the insert of Figure 6C, which are 0.25 and 0.9 emu/g for CMNPs and 13.2 and 12.9 Oe for MLs, respectively. Superparamagnetic properties could be suggested for CMNPs and MLs, with the remnant magnetization being close to zero and the coercivity being low from the magnetization curve. Combined with the sufficient magnetization value, MLs will be bestowed with the critical properties required for magnetically targeted cancer therapy.

The results from thermogravimetric analysis (TGA) of blank liposomes, CMNPs, and MLs are shown in Figure 6D, together with the differential thermal analysis (DTA) curves shown in the figure insert to elucidate the thermal properties and loading percentage of CMNPs in liposomes. The early weight loss before 200 °C for all samples may be assigned to the losses of residually bound water and absorbed CO_2_. For CMNPs, some weight loss was recorded starting from ~300 °C, reaching a final residual weight of ~94% (*w/w*) at 650 °C from the thermal decomposition of coated citric acid on the surface of CMNPs [47]. For blank liposomes, substantial weight loss occurs within 260 °C to 380 °C, with a sharp decomposition peak temperature at ~360 °C and a shoulder peak temperature at ~290 °C shown from the DTA curve. The residual weight of blank liposomes was 10.3% (*w*/*w*) at the end of temperature increase to 650 °C. In comparison, MLs displayed a similar decomposition peak, temperature, but the shoulder temperature transformed into a more distinctive peak temperature possibly due to the interactions of lipids with CMNPs [48]. From the residual weight difference between MLs and blank liposomes at 650 °C, the weight percentage of CMNPs in MLs was estimated to be ~21.0% (*w*/*w*), assuming negligible weight loss for CMNPs, which compared favorably with that obtained from chemical analysis (21.8% ± 1.7%).

### 2.3. Heating Efficiency Induced by AMF, NIR, or Combined Laser Treament

**Figure 7:**
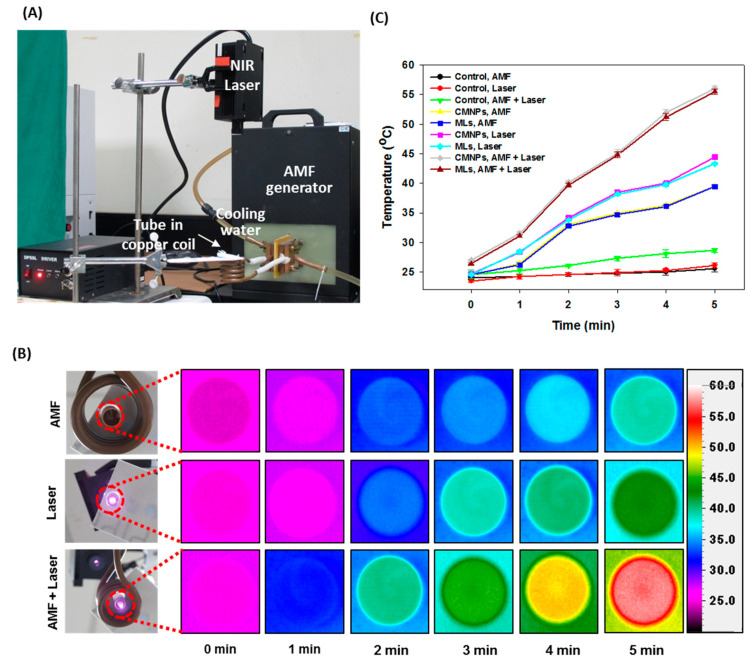
In vitro heating efficiency of CMNPs and MLs as induced by magnetic hyperthermia (MH) or photothermia (PT). A 500 μL solution of CMNPs or MLs prepared in distilled water (corresponding to 0.6 mg/mL CMNPs) was taken in a 2 mL Eppendorf tube and subject to AMF or NIR laser treatment. The tube was placed in the center of a 30.5 mm internal diameter solenoid copper coil for AMF induction at 52 kHz or 808 nm NIR laser exposure at 1.8 W/cm^2^ from the top (**A**). The time-lapsed thermal images were acquired from tube bottom with an infrared thermal camera (**B**) and the peak temperature was plotted as a function of treatment time (**C**). The control was distilled water without CMNPs.

The heating efficiency study was carried out by treating a suspension of MLs or CMNPs in water with AMF, NIR laser, or combined AMF + NIR laser treatment to examine whether dual MH–PT could amplify the heating power of MLs. For this purpose, samples were prepared in distilled water in Eppendorf tubes and subjected to AMF, NIR, or combined laser treatment up to 5 min (Figure 7A). The thermal images of the solutions were captured from the bottoms of the tubes with an infrared thermal camera (Figure 7B). The peak temperatures acquired from the time-lapsed thermal images were plotted vs. time to compare the heating efficiency subject to different treatments (Figure 7C). A fixed base concentration of CMNPs (0.6 mg/mL) was used to compare the heating efficiency between CMNPs and MLs. There was no statistical difference in temperature change between MLs and CMNPs regardless of mode of treatment, indicating encapsulation of CMNPs in MLs did not influence the heating efficiency at the same concentration of heating agent in the solution. For AMF treatment at 52 kHz frequency for 5 min, the temperature reached 39 °C. The samples exposed to NIR laser (808 nm) for 5 min at 1.8 W/cm^2^ showed better heating efficiency compared with AMF, where the temperature reached 43 °C. This behavior is consistent with a recent report that PT is far more efficient than MH using magnetite nanoparticles [49]. Most importantly, in comparison to single-mode operation employing AMF or NIR lasers, dual-mode operation employing AMF + NIR lasers provides better heating efficiency, as the temperature reaches 56 °C at the end of the same treatment period. Taken together, the in vitro heating experiments support that CMNPs alone can increase the temperature from dipole interactions under AMF and with the absorbance of light near the NIR region [50]. Nonetheless, the combined use of AMF and NIR lasers could enhance the heating efficiency of MLs originated from encapsulated CMNPs. By taking advantage of the combined effect of dual-mode treatment, it is expected that the efficacy of cancer hyperthermia treatment could be enhanced by using a lower concentration of the heating agent and a shorter treatment time [51].

For quantitative comparison, the specific absorption rate (SAR, W/g CMNPs) was determined from the slope of each heating curve in Figure 7C by linear regression of all data points in a single curve, using the specific heat capacity of water and concentration of CMNPs [52,53]. As shown in Table 4, there is no significant difference in SAR between CMNPs and MLs (based on unit weight of CMNPs) regardless of the stimulus modality, supporting the observation that CMNPs in MLs could fully preserve the response to MH–PT. Comparing the single modality, PT shows significant improvement of SAR compared to MH. Indeed, dual AMF–NIR laser stimulus modality resulted in elevated SAR values, which were 2.0 and 1.5 times greater than that of AMF and NIR lasers alone, respectively.

### 2.4. Intracellular Uptake of MLs

**Figure 8:**
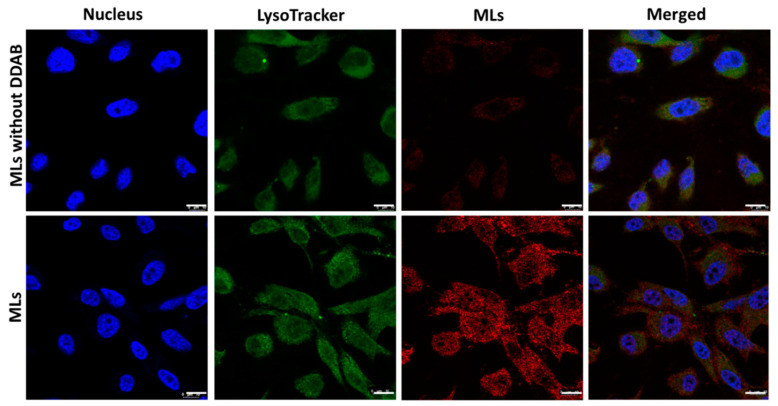
Intracellular uptake of MLs and control liposomes (MLs prepared without cationic lipid DDAB) by U87 cancer cells. The IR 780-labelled liposomes (red) were incubated with U87 cells for 24 h. Lysosomes and cell nuclei were stained separately with LysoTracker (green) and DAPI (blue) and observed by confocal laser scanning microscopy. Bar = 10 μm.

Cationic liposomes have the potential to carry cargos (such as drugs) and accumulate in tumor tissues owing to their positive charge [54]. This passive accumulation process can result in significant accumulation of loaded drugs compared with the administration of free drugs [55]. Due to their targeted and drug release functions, cationic liposomes show the potential to increase drug efficacy in tumors [56]. Considering the positive zeta potential of MLs (Table 3) originated from the cationic lipid DDAB in the lipid composition, we expected the designed MLs could be able to selectively bind onto the surfaces of U87 glioblastoma cells, thus facilitating cellular trafficking through charge-mediated endocytosis for enhanced therapeutic effects from the thermally induced killing of cancer cells. The increased intracellular uptake of MLs as induced by the targeting function of the cationic MLs was confirmed from confocal laser scanning microscopy and compared with control MLs (MLs prepared without cationic lipid DDAB) (Figure 8). The confocal microscopy images revealed intracellular red fluorescence corresponding to IR780-labeled MLs when U87 cells were exposed to MLs for 24 h. This illustrates endocytosis and subsequent accumulation of MLs in the cell cytoplasm after internalization. MLs (red fluorescence) were observed to be internalized in U87 cells and appeared to be co-localized with lysosomes stained with LysoTracker (green fluorescence). The contributing factor may be related to the intracellular localization of MLs in the acidic compartment in the cytosol. As with the merged images in Figure 8, only MLs but not control liposomes showed high fluorescence intensity (yellow) corresponding to both MLs (red) and LysoTracker (green), indicating efficient uptake of MLs through endocytosis. Besides lysosomal co-localization, MLs not co-localized with LysoTracker were also observed to a large extent. This is consistent with the finding that cationic liposome can undergo electrostatic-interaction-mediated membrane fusion with the anionic endosome membrane, thus escaping the endosome [57]. Therefore, co-staining of the acidic organelles (lysosomes) demonstrated that despite not all MLs being associated with lysosomes, an important proportion of them did end up in this acidic cell compartment.

Compared to U87 cells treated with MLs, a drastically diminished intracellular red fluorescence signal was observed for control MLs prepared without DDAB (Figure 8). This difference is due to the contribution of DDAB, which interacts with the cell membrane for passive targeting to facilitate intracellular uptake. As the U87 cell surface is negatively charged, the DDAB-containing cationic MLs rapidly interact with biological membranes to allow for facilitated passive targeting and particle internalization, in comparison with control liposomes [58]. This induced specific cellular targeting and uptake of MLs will lead to an enhanced MH–PT effect for increased cytotoxicity and provide an efficient milieu for thermally induced cell death in vitro.

### 2.5. Thermally Induced Cancer Cell Killing In Vitro

**Figure 9:**
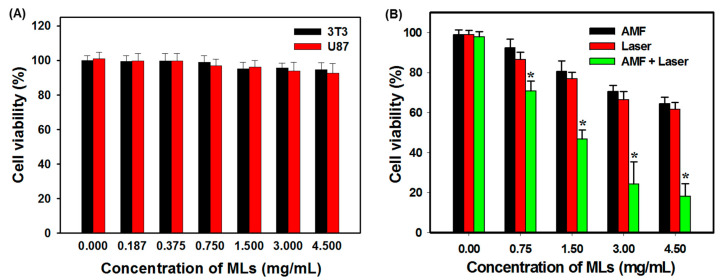
(**A**) Biocompatibility of MLs toward U87 and 3T3 cells without thermal induction. (**B**) Thermally induced killing of U87 cells by MLs after induced heating by AMF or NIR lasers. Note: * *p* < 0.05 compared with AMF and NIR lasers. The cell viability was compared to the cell culture medium without MLs using MTT assays.

After confirming the intracellular uptake, the biocompatibility of MLs was studied by exposing U87 (tumor) and 3T3 (normal) cells to various concentrations of MLs without thermal induction. As shown in Figure 9A, the relative cell viability (compared to cell culture medium) remained above 95% for MLs concentrations up to 4.5 mg/mL (in cell culture medium), confirming that the cell viability was not affected by internalization of MLs and that the prepared MLs appear to be biocompatible. The thermally induced cancer cell killing effect was examined by incubating U87 with different concentrations of MLs, followed by AMF or NIR laser treatment. As showed in Figure 9B, dose-dependent cytotoxicity was observed when using MLs as a thermal inductive agent for killing U87 cancer cells after subject to MH or PT treatment. There was no significant difference in cytotoxicity between the AMF and NIR laser groups, although dosage-dependent cytotoxicity occurred at higher concentrations of MLs. Most importantly, AMF + NIR laser dual-mode treatment led to a significant reduction in cell viability compared with single-mode AMF or NIR laser treatments. At the maximum concentration of 4.5 mg/mL, AMF and NIR lasers reduce cell viability by 32–35%, which could be significantly enhanced by 2.4-fold to 82% for AMF + NIR lasers. These outcomes demonstrated the synergistic impact with an improved thermal effect induced by magneto-photothermal treatment [17].

**Figure 10:**
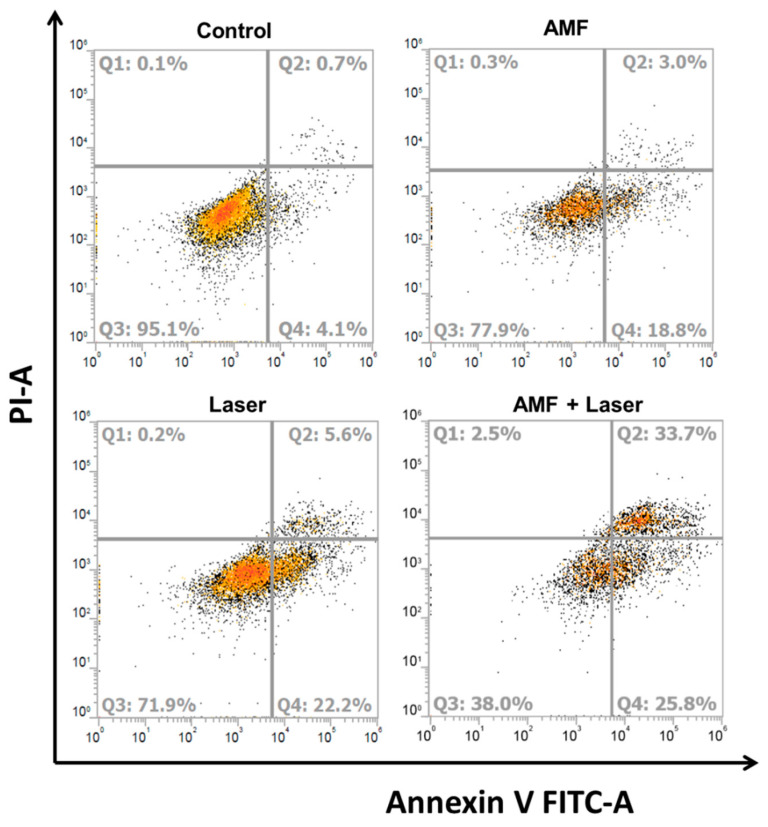
The flow cytometer analysis of the apoptotic and necrotic cells by Annexin V-FITC/PI staining (Q1: necrotic; Q2: late apoptotic; Q3: live; Q4: early apoptotic) after incubating U87 cells with MLs (2 mg/mL) and subjecting them to thermally induced cell killing by AMF or NIR laser. The control is without any treatment.

We further evaluated the cytotoxicity of MLs after AMF and NIR laser treatments using flow cytometry analysis. The cells were stained with Annexin V/PI to examine the percentage of live (Q3), early apoptotic (Q4), late apoptotic (Q2), and necrotic cells (Q1) according to differences in plasma membrane permeability and integrity (Figure 10). From Table 5, the flow cytometry data confirm that cell death occurred primarily from apoptosis associated with MH [59] or PT [60]. Similar to the result obtained from the MTT assay (Figure 9A), the control group without any treatment shows a minimum apoptotic rate with >95% viable cells. Single-mode treatment with AMF (or NIR laser) resulted in ~19% (~22%) early apoptosis and ~3% (~6%) late apoptosis. With AMF treatment, MH resulted in cell death and the live cell percentage decreased to 77.9%. With NIR laser treatment, a more pronounced cell apoptosis rate was observed due to higher thermal effects induced by PT (Figure 7C), while the live cell percentage decreased to 71.9%. With combined AMF + NIR laser treatment, ~26% early apoptosis and ~34% late apoptosis rates were observed. The dual treatment also revealed a slightly increased necrosis rate not observed from single treatment and gave the most pronounced decrease of live cell percentage to 38%, due to combined MH–PT.

The in vitro cytotoxicity study, thus, suggests AMF–NIR laser treatment could induce dual hyperthermia modality for future translational study in vivo. This proof-of-concept study in vitro should endorse future clinical application of the optimized cationic MLs developed in this study. The high EE of CMNPs and their suitable size for internalization by the targeted cancer cells could facilitate the use of MLs developed here for dual MH–PT cancer therapy.

## 3. Materials and Methods

### 3.1. Materials

The 1,2-Dipalmitoyl-sn-glycero-3-phosphocholine (DPPC) was purchased from Avanti Polar Lipids (Alabaster, AL, USA). Dimethyldioctadecyl ammonium bromide (DDAB), cholesterol (CH), Triton X-100, and IR-780 iodide were acquired from Sigma-Aldrich (St. Louis, MO, USA). Fe(II) chloride tetrahydrate (99%) and Fe(III) chloride hexahydrate (97%) were purchased from Acros Organics (Geel, Belgium). Cell culture reagents were purchased from Life Technologies (Carlsbad, CA, USA). All chemicals and reagents were of analytical grade and were used as received.

### 3.2. Synthesis of Citric-Acid-Coated Iron Oxide Magnetic Nanoparticles (CMNPs)

The CMNPs were synthesized according to the procedure reported in previous studies [20,32]. Briefly, FeCl_2_ and FeCl_3_ at a 1:2 molar ratio were taken in a three-neck flask and dissolved with 40 mL distilled deionized water (DDI water). The solution was mixed at 100 rpm with nitrogen purging for 15 min at 70–80 °C, followed by adding 5 mL NH_4_OH (25%) to maintain the solution at pH 10 under vigorous stirring for 60 min. For the citric acid coating, the solution temperature was increased to 95 °C, citric acid (1 mg/mL) was added in a dropwise manner, and the reaction was continued for another 90 min. The formed CMNPs were purified by dialysis against DDI water using a 3.5 kDa molecular weight cut-off (MWCO) dialysis membrane.

### 3.3. Preparation of Magnetic Liposomes (MLs)

The CMNP-encapsulated MLs were prepared using the thin-film hydration and extrusion method [61,62]. Briefly, solutions of DPPC/DDAB/CH (according to experimental design) with various molar ratios were prepared in a mixed solvent system of ethanol and chloroform (1:2, *v/v*) in a round-bottom flask. The organic solvents were evaporated in a rotary evaporator (EYELA N-1200AVF, Tokyo, Japan) by rotating the flask in a 50 °C water bath at 100 psi for 30 min. The thin film formed on the wall of the flask was further dried in a vacuum oven overnight to remove excess organic solvents. A pH 7.4 phosphate buffer solution (10 mM) containing CMNPs of various concentrations (according to experimental design) was used to hydrate the lipid thin film for 30 min, then the solution was sonicated for 30 min. A 0.2-μm polycarbonate membrane (Whatman, NJ, USA) was used to extrude the MLs for 15–20 cycles in a commercial extruder (Lipex extruder, Transferra Nanosciences, BC, Canada). To remove CMNPs not incorporated in MLs, the solution was centrifugation at 1000 g for 15 min, then MLs were collected in the supernatant [63]. For physico-chemical characterization, blank liposome was prepared similarly using the optimized formulation of MLs but without CMNPs. For comparison of the intracellular uptake, control liposome was prepared similarly using the optimized formulation of MLs but without DDAB. The amount of CMNPs in MLs was determined by spectrophotometric analysis [64]. Briefly, 5 μL of Triton X-100 (1%, *v*/*v*) was added to 20 μL of MLs and incubated at 43 °C for 10 min to break the liposomes. After adding 225 μL HCl (37%), 250 μL 40 mM KSCN, and incubating the solution for 5 min, the absorbance of the solution was measured at 480 nm by a UV-VIS spectrophotometer (GENESYS 150, Thermo Fisher Scientific, Waltham, MA, USA). The concentration of CMNPs was then calculated from a pre-determined standard curve. The encapsulation efficiency (EE) of CMNPs in MLs was calculated using the following equation.

(3) $$\mathrm{EE}\;\left(\%\right)=\frac{\mathrm{Weight}\;\mathrm{of}\;\mathrm{CMNPs}\;\mathrm{encapsulated}\;\mathrm{in}\;\mathrm{MLs}}{\mathrm{Weight}\;\mathrm{of}\;\mathrm{CMNPs}\;\mathrm{used}\;\mathrm{in}\;\mathrm{preparation}\;\mathrm{of}\;\mathrm{MLs}}\times 100$$

### 3.4. Experimental Design

The response surface methodology (RSM) is a conventional method for experimental design, which was applied in this study to optimize the preparation of MLs [32,65]. The experiment is designed based on four-factor and five-level central composite rotatable design (CCRD), which includes 16 factorial points, 7 replicates at center points, and 8 axial points, with a total of 31 experimental runs. The effects of four independent variables (mass of DPPC, mass of DDAB, mass of CH, and concentration of CMNPs, coded as X_1_, X_2_, X_3_ and X_4_, respectively) were identified as key factors responsible for the EE and liposome size. Based on preliminary experiments, the ranges of the factors were chosen as 1 to 5 µmol for X_1_; 1 to 3 µmol for X_2_ and X_3_; 0.1 to 1.1 mg/mL for X_4_ (Table 6). As indicated by the model, a response surface could be identified with the chosen variables using a second-order polynomial model:

(4) $$Y_{i}=\alpha_{0}+\sum_{i}\alpha_{i}X_{i}+\sum_{i}\alpha_{\mathrm{ii}}X_{i}^{2}+\sum_{i\neq j}\alpha_{\mathrm{ij}}X_{i}X_{j}$$

where X_i_ and X_j_ are the coded values of independent variables; Y_i_ is the predicted response; α_0_ is the intercept coefficient; and α_i_, α_ii_, and α_ij_ are the linear coefficients, squared coefficients, and interaction coefficients, respectively. Each experiment was performed in duplicate, except at the central point. The fitting quality of the model was assessed by the coefficients of determination (R^2^) and the analysis of variance (ANOVA). All calculations were carried out using the Statistica software (TIBCO Software, Palo Alto, CA, USA).

### 3.5. Charactrization of Physico-Chemical Properties

The size distribution and the zeta potential of CMNPs and MLs were measured by dynamic light scattering (DLS) using a Nano ZS Zetasizer (Malvern Instruments Ltd., Worcestershire, UK) at 25 °C and a scattering angle of 173° in auto mode. The samples were dilute with DDI water for DLS and with 10 mM pH 7.4 phosphate buffer for zeta potential measurements. A JEOL JEM-2000-EX II transmission electron microscope (TEM) was used to examine the size of CMNPs at 100 kV. A cryo-TEM (JEOL JEM-1400, Tokyo, Japan) was used to observe the size and morphology MLs after drop-casting MLs onto a 300 mesh carbon-coated copper grid. By plunge-freezing the sample, the fixed specimen was mounted for observation at 120 kV and −170 °C. A Bruker TENSOR II Fourier transform infrared (FTIR) spectrometer (Bruker Optics, Inc., Billerica, MA, USA) was used for FTIR spectroscopy after blending the sample with KBr for analysis from 400 to 4000 cm^−1^ at 4 cm^−1^ resolution and 2.5 mm/s scanning rate. The magnetization properties were determined using a superconductive quantum interference device (SQUID) magnetometer (MPMS XL-7, Quantum Design, San Diego, CA, USA) from −10,000 to +10,000 G applied magnetic fields at 25 °C. The samples were freeze dried before SQUID measurements. Themogravimetric analysis (TGA) was performed with 10 mg dried powder samples in nitrogen atmosphere from 100 to 650 °C using a Q50 TGA from TA Instruments (New Castle, DE, USA). The heating rate and nitrogen flow rate were maintained at 10 °C/min and 60 mL/min, respectively. X-ray diffraction (XRD) was performed using a D_2_ Phaser X-ray powder diffractometer (Bruker, Madison, WI, USA) with a scanning range (2θ) of 20° to 70° with Cu Kα radiation. The crystalline size was calculated using the Debye–Scherrer equation at the strongest peak and the phase was compared with the existing JCPDS database.

(5) $$D=\frac{\kappa \times \lambda }{\beta \times \cos\theta }$$

where D is the average crystalline domain size (nm), λ is the wavelength of X-ray (nm), κ is a factor (~0.9), β is the peak width at half maxima height intensity, and θ is the Bragg angle.

The stability of MLs was studied using nanoparticle tracking analysis (NTA) for simultaneous determination of the particle size distribution and particle concentration using a NanoSight LM10 instrument (Malvern Panalytical, Malvern, UK) equipped with a 405 nm laser. A 3 mg/mL MLs solution prepared in phosphate-buffered saline (PBS) was incubated statically at 37 °C. At different time points, samples were removed and analyzed by NTA at room temperature. The samples were measured for 60 s with manual gain and shutter adjustments.

### 3.6. Heating Efficiency Induced by AMF and NIR Lasers

To evaluate the heating ability, time-lapsed thermal images of CMNPs and MLs solutions were captured with an infrared thermal camera (InfReC Thermo GEAR G100EX, Nippon Avionics Co., Tokyo, Japan) after AMF, NIR, or AMF + NIR laser treatments, from which peak temperature profiles could be obtained. A 500 µL suspension of CMNPs or MLs (corresponding to 0.6 mg/mL CMNPs) prepared in distilled (DI) water was taken in a 2 mL Eppendorf tube. For AMF treatment, the tube was placed in the center of a solenoid copper coil (inner diameter = 30.5 mm, 5 turns) for magnetic hyperthermia (MH) at 52 kHz while maintaining the temperature of the copper coil at 37 °C with circulating cooling water. For the NIR laser treatment, the tube was exposed to an 808 nm laser 1.8 W/cm^2^ from the top for photothermia (PT). For the AMF + NIR laser treatment, the tube was placed in the center of a solenoid copper coil (inner diameter = 30.5 mm, 5 turns) at 52 kHz frequency while simultaneously exposed to an 808 nm laser 1.8 W/cm^2^ from the top for MH–PT. The thermal images were acquired from the bottom of the tube with an infrared thermal camera. The specific absorption rate (SAR) of CMNPs and MLs based on the weight of CMNPs was calculated from the temperature vs. time curve:

(6) $$SAR=\frac{C}{m}\frac{\Delta T}{\Delta t}$$

where *C* is the specific heat capacity of water, ΔT/Δt is the slope of the recorded temperature increase, and *m* is the concentration of CMNPs used.

### 3.7. Intracellular Uptake of MLs by Cancer Cells

The intracellular uptake of MLs was studied with U87 human primary glioblastoma cells (ATCC HTB1) obtained from the American Type Culture Collection (Manassas, VA, USA). The cells were seeded on a 15 mm coverslip in a 24-well culture plate at 5 × 10^4^ cells per well and cultured in cell culture medium (Dulbecco’s modified Eagle’s medium (DMEM) supplemented with 10% fetal bovine serum) for 24 h in a humidified CO_2_ incubator at 37 °C under 5% CO_2_. After washing with PBS, the medium was replaced with IR-780-labeled MLs prepared in cell culture medium (0.5 mg/mL) and cultured for 24 h. The labeling of MLs was achieved by encapsulating IR-780 dye in the aqueous cores of the liposomes. By following the same procedure as used to prepare MLs, the dried lipid film was hydrated with a solution containing CMNPs and IR-780 iodide for 30 min at 43 °C. The excess IR-780 was removed by dialysis with 12–14 kDa MWCO dialysis membranes in PBS for 24 h. The sample was ultra-centrifuged at 64,000× *g* for 30 min and the pellet was re-suspended with cell culture medium. After incubation with IR-780-labeled MLs for 24 h, the cells were washed with PBS and lysosomes were labeled with LysoTracker Green DND-26 (1 µM, Thermo Fisher Scientific, Waltham, MA, USA) for 60 min at 37 °C. The labeled cells were washed twice with PBS and fixed with 4% paraformaldehyde, then further treated with Triton X-100 (0.1% in PBS). Finally, the cells were counterstained with Hoechst 33342 (1 µg/mL, Thermo Fisher Scientific) for cell nucleus (blue) assessment for 15 min. The coverslip was removed and mounted on a glass slide for confocal laser scanning microscopy analysis. Identification of liposomes was rendered possible by red fluorescence signals from IR-780-labeled MLs. Cellular liposomal internalization was measured through a LSM 510 Meta inverted confocal microscope (Zeiss, Oberkochen, Germany) with an excitation/emission wavelengths of 350/451, 504/511, and 684/784 nm for blue, green, and red, respectively.

### 3.8. In Vitro Biocompatibility of MLs

To study the biocompatibility of MLs, 2.5 × 10^3^ U87 cells or 3T3 fibroblasts was seeded into each well of a 96-well cell culture plate and cultured in cell culture medium (Dulbecco’s modified Eagle’s medium (DMEM) supplemented with 10% fetal bovine serum) overnight in a humidified CO_2_ incubator at 37 °C under 5% CO_2_. After removing the culture medium, a test sample containing 0.18 to 4.5 mg/mL MLs in cell culture medium was added to each well and cultured for another 24 h at 37 °C. The viability of the cells was measured by the 3-[4,5-dimethylthiazol-2-yl]-2,5 diphenyltetrazolium bromide (MTT) assay at 540 nm with a microplate reader. The relative cell viability (%) was determined, with the viability of cells incubated with culture medium without particles taken as 100%.

### 3.9. In Vitro Cancer Cell Killing by AMF and NIR Lasers

To study the cytotoxicity in the presence of AMF, NIR, or AMF + NIR laser treatments, 5 × 10^4^ U87 cells were seeded to each well of a six-well cell culture plate and cultured overnight at 37 °C under 5% CO_2_. After removing the culture medium, a 500 µL test sample containing MLs in cell culture medium was added to a final concentration of 0.75 to 4.5 mg/mL and cultured at 37 °C for 24 h. The cells were detached from the well surface after treatment with 0.1% trypsin/EDTA and centrifuged. The cell pellet was re-dispersed with 500 µL fresh cell culture medium and placed in a 2 mL Eppendorf tubes for subsequent treatment. For AMF treatment, the Eppendorf tube containing cells was placed in the center of a copper coil (inner diameter = 30.5 mm, 5 turns) at 52 kHz for 5 min. For NIR laser treatment, the Eppendorf tube containing cells was exposed to a NIR 808 nm laser at 1.8 W/cm^2^ for 5 min. For the AMF + NIR laser treatment, the Eppendorf tube was placed in the center of the copper coil at 52 kHz while simultaneously exposed to a NIR 808 nm laser at 1.8 W/cm^2^ for 5 min for MH–PT. At the end of treatment, cells in each tube were transferred back into a well in a new 6-well culture plate, replenished with fresh cell culture medium, and cultured for 24 h at 37 °C and 5% CO_2_. The relative cell viability (%) was determined. The relative cell viability (%) was determined by the MTT assay in the same manner as before, with the viability of cells incubated with culture medium without particles taken as 100% [45].

### 3.10. Flow Cytometry Analysis for Apoptosis and Necrosis

To study the apoptosis and necrosis in U87 cells, 5 × 10^5^ cells were seeded in T-25 cell culture flasks and incubated in a humidified CO_2_ incubator at 37 °C under 5% CO_2_. After 24 h incubation, the cells were washed with PBS and incubated with MLs in cell culture medium (2 mg/mL). Later, the cells were trypsinized and collected in 500 µL cell culture medium. The cell suspension was placed in a 2 mL Eppendorf tube and treated with AMF and NIR lasers at 52 kHz (AMF) and 1.8 W/cm^2^ (NIR laser) in the same manner as before for 5 min. After adding 500 µL fresh medium, the cell suspension was reacted with fluorescein isothiocyanate-labeled Annexin V (FITC-Annexin V) for 30 min followed by propidium iodide (PI) for flow cytometry analysis (Attune NxT flow cytometer, Thermo Fisher Scientific, Waltham, MA, USA).

### 3.11. Statistical Analysis

All data are presented as the mean ± standard deviation (SD) and were subjected to one-way analysis of variance (ANOVA) with post hoc Tukey’s HSD test using IBM SPSS statistics software. Differences were considered to be significant at *p* < 0.05.

## 4. Conclusions

Using the thin-film hydration method, we successfully optimized the preparation of cationic MLs, using the EE of CMNPs and size of MLs as the responses. The developed RSM model was verified to be consistent with the experiment values using the optimized variables. Physico-chemical characterization confirmed the successful synthesis of CMNP and MLs through the high suspension stability of MLs. In vitro thermal effects of MLs supported by the dual-mode AMF + NIR laser operation could enhance the thermal induction efficiency of MLs by combining MH and PT. With the efficient intracellular uptake by and high biocompatibility toward U87 cancer cells, MLs were tested for dual-mode thermal therapy using AMF and NIR lasers. The enhanced hyperthermia effects for cancer cell killing as demonstrated from MTT assays and flow cytometry analysis support the combined use of AMF and NIR lasers as an effective hyperthermia treatment for cancer cells in vitro. By combining MH–PT dual thermal treatments induced by MLs, this approach could overcome some of the individual deficiencies to achieve the most promising cancer cell killing effect. Thus, this proof-of-concept in vitro study clearly indicates that the cationic MLs developed in this study will be a potential candidate for future bimodal magneto-photothermal cancer therapy in vivo.

## Figures and Tables

**Table 1:** Central composite design arrangement and observed response.

Design Points	Coded Independent Variables				Response Values	
	X_1_	X_2_	X_3_	X_4_	EE (%)	Size (nm)
1	−1	−1	−1	−1	23.6	135.2
2	1	−1	−1	−1	31.3	153.0
3	−1	1	−1	−1	65.3	183.5
4	1	1	−1	−1	65.6	170.1
5	−1	−1	1	−1	22.1	132.4
6	1	−1	1	−1	28.8	152.8
7	−1	1	1	−1	48.4	180.8
8	1	1	1	−1	30.0	138.9
9	−1	−1	−1	1	45.9	155.5
10	1	−1	−1	1	19.5	126.1
11	−1	1	−1	1	24.7	148.7
12	1	1	−1	1	21.9	179.6
13	−1	−1	1	1	44.3	143.6
14	1	−1	1	1	18.3	147.1
15	−1	1	1	1	43.6	147.8
16	1	1	1	1	43.0	130.8
17	−2	0	0	0	38.0	164.8
18	2	0	0	0	29.1	138.6
19	0	−2	0	0	19.2	134.8
20	0	2	0	0	44.3	157.5
21	0	0	−2	0	34.5	206.3
22	0	0	2	0	48.1	144.2
23	0	0	0	−2	21.2	172.7
24	0	0	0	2	25.9	127.0
25	0	0	0	0	19.5	142.9
26	0	0	0	0	23.6	119.6
27	0	0	0	0	14.9	122.5
28	0	0	0	0	19.6	124.8
29	0	0	0	0	24.8	127.0
30	0	0	0	0	26.7	133.1
31	0	0	0	0	28.1	129.7

**Table 2:** Validation of the model with predicated experimental values.

			Predicted Values		Experimental Values	
Factors ^1^	Codes	Variables	EE (%) ^2^	Size (nm) ^3^	EE (%) ^2^	Size (nm) ^3^
DPPC	X_1_	2 μmol	86.9	121.1	84.2 ± 4.7	124.3 ± 6.5
DDAB	X_2_	0.5 μmol				
CH	X_3_	1 μmol				
CMNP	X_4_	0.25 mg/mL				

^1^ DPPC: 1,2-dipalmitoyl-sn-glycero-3-phosphocholine, DDAB: dimethyldioctadecyl ammonium bromide, CH: cholesterol, CMNP: citric-acid-coated magnetic nanoparticles. ^2^ EE: encapsulation efficiency. ^3^ Size: size of magnetic liposomes from dynamic light scattering.

**Table 3:** Size and zeta potentials values.

Samples ^1^	Size from DLS (nm) ^2^	PDI ^3^	Zeta Potential (mV)
CMNPs	36.5 ± 5.4	0.21 ± 0.06	−19.4 ± 4.3
MLs	124.3 ± 6.5	0.28 ± 0.09	16.3 ± 3.7

^1^ CMNPs: citric-acid-coated iron oxide magnetic nanoparticles, MLs: magnetic liposomes. ^2^ DLS: dynamic light scattering. ^3^ PDI: polydispersity index.

**Table 4:** Specific absorption rates (SARs) of CMNPs and MLs at 0.6 mg/mL CMNP equivalent ^1^.

Sample ^2^	AMF ^3^	Laser	AMF + Laser
CMNPs	21.20 ± 0.14	28.06 ± 0.21 *	42.13 ± 0.58 *^,#^
MLs	21.08 ± 0.16	28.12 ± 0.17 *	42.04 ± 0.48 *^,#^

^1^ SAR = (C/m) (ΔT/Δt), where C is the specific heat capacity of water, ΔT/Δt is the slope of the temperature vs. time curve, m is the concentration of CMNPs used. ^2^ CMNPs: citric-acid-coated iron oxide magnetic nanoparticles; MLs: magnetic liposomes. ^3^ AMF: alternating magnetic field; * *p* < 0.05 compared with AMF; ^#^ *p* < 0.05 compared with Laser.

**Table 5:** Apoptotic and necrotic analysis from flow cytometry analysis.

	Control	AMF ^1^	Laser	AMF + Laser
Live cell (Q3)	95.1%	77.9%	71.9%	38.0%
Early apoptosis (Q4)	4.1%	18.8%	22.2%	25.8%
Late apoptosis (Q2)	0.7%	3.0%	5.6%	33.7%
Necrotic cell (Q1)	0.4%	0.3%	0.2%	2.5%

^1^ AMF: alternating magnetic field.

**Table 6:** The central composite design showing the independent variables and levels used in the experiments.

Factors	Codes	Variable Levels				
		2	−1	0	−1	−2
DPPC (µmol)	X_1_	1	2	3	4	5
DDAB (µmol)	X_2_	1	1.5	2	2.5	3
CH (µmol)	X_3_	1	1.5	2	2.5	3
CMNPs (mg/mL)	X_4_	0.1	0.35	0.6	0.85	1.1
